# Assessing the Diets of Young Children and Adolescents in India: Challenges and Opportunities

**DOI:** 10.3389/fped.2022.725812

**Published:** 2022-05-17

**Authors:** Lindsey M. Locks, Miloni Shah, Shilpa Bhaise, Patricia L. Hibberd, Archana Patel

**Affiliations:** ^1^Department of Health Sciences, College of Health and Rehabilitation Sciences: Sargent College, Boston University, Boston, MA, United States; ^2^Department of Global Health, School of Public Health, Boston University, Boston, MA, United States; ^3^Lata Medical Research Foundation, Nagpur, India; ^4^Department of Infectious Diseases, School of Medicine, Boston University, Boston, MA, United States; ^5^Datta Meghe Institute of Medical Sciences, Sawangi, India

**Keywords:** diet, children, adolescents, India, nutrition

## Abstract

Sustainably addressing the crisis of undernutrition for children and adolescents in underserved and resource-limited communities will require, among other investments, interventions aimed at optimizing the diets of these vulnerable populations. However, to date, there are substantial global gaps in the collection of dietary data in children and adolescents. This review article summarizes the challenges and opportunities in assessing diet among children and adolescents in India. National surveys in India identify the scale of the triple burden of malnutrition (undernutrition, micronutrient deficiencies and overnutrition) in children and adolescents and assess key nutrition and food security indicators for making informed policy decisions. However, national surveys do not collect data on diet, instead relying on anthropometry, biomarkers of micronutrient deficiencies, and summary measures of diet, such as the WHO infant and young child feeding summary indicators. Sub-national surveys and the scientific literature thus fill important gaps in describing the nutrient intakes of children and adolescents in India; however large gaps remain. Future research can be improved by investments in infrastructure to streamline the assessment of diet in India. The current challenges confronting the collection and analysis of high-quality dietary data occur in both the data collection and data analysis phases. Common methods for assessing diets in low-resource settings—such as 24 h recalls and food frequency questionnaires are particularly challenging to implement well in young children and adolescents due to motivation and memory issues in young respondents. Additionally, there are challenges with parental recall including children having multiple caretakers and meals outside the home. Furthermore, analysis of dietary data is hindered by the lack of affordable, accessible software for dietary data analysis relevant to the diversity in Indian diets. New technologies can address some of the challenges in dietary data collection and analysis, but to date, there are no platforms designed for population-level dietary assessment in India. Public and private sector investment in dietary assessment, as well as collaboration of researchers and the creation of open-source platforms for the sharing of data inputs (local food lists, recipe databases, etc.) will be essential to build infrastructure to better understand the diets of children and adolescents in India and improve dietary interventions in these target groups.

## Introduction

Poor diet quality is one of the leading causes of ill health globally (1, 2). A healthy diet is essential across all stages of the life cycle; however, children and adolescents have unique nutritional needs. Childhood and adolescence are periods of rapid growth and development. It is also when dietary preferences and habits form that may last a lifetime (3, 4). Not only do the nutritional requirements and recommended eating practices change throughout childhood and adolescence, but so do the socio-ecological factors that affect their diets. While the meals of infants and young children are predominantly consumed with parents and caretakers, as children age, exposures such as the school food environment, advertising and peer pressure become increasingly more important. India currently faces the triple burden of malnutrition—with high rates of undernutrition (stunting, wasting & underweight), micronutrient deficiencies, overweight and obesity (5). Effectively managing the triple burden of malnutrition will require holistic, integrated programs that improve the diet quality of India's youth and are first able to effectively measure diet quality in children and adolescents. However, there is a global dearth of high-quality data on dietary intake in these populations, particularly in Low and Middle Income Countries (LMICs) (6, 7). Although national surveys in India include essential nutrition information—such as the prevalence of stunting, wasting, overweight and anemia, they do not currently summarize overall diet quality. India has 29 states and 9 union territories with diverse diets. Each state, and regions within each state, have different culinary traditions and food quality varies across the country. Research on dietary intake in India is thus hindered, by the country's diversity, limited expertise in dietary assessment across regions, and the high-cost and time burden of collecting high-quality dietary data.

Dietary surveys in children and adolescent that include appropriate dietary assessments are essential for the development of effective policies and programs aimed at optimizing eating behaviors among India's youth. This landscape analysis emerges from a review of the following topics that help to understand and inform the nutritional needs of children and adolescents with a focus on dietary assessments. They are 1) global and national dietary recommendations for children and adolescents; 2) existing national and sub-national surveys that assess nutrition, as well as the scientific literature that specifically measures diet in India; 3) we describe commonly used methods for assessing diet; 4) the emergence of novel technology that can be harnessed for large-scale dietary data collection and analysis. The review concludes with recommendations that may help streamline and improve research on the diet of children and adolescents in India.

## Methods

The purpose of this review was to summarize research methodologies used for collecting dietary data for children and adolescents in India. In addition, the focus of this review was to understand the challenges and opportunities in conducting dietary assessment. Accordingly below mentioned steps were followed.

### Search Strategy

We searched the databases PubMed, Web of Science, and Embase on March 31, 2020 and included all articles published and indexed by that date. From PubMed, 62 articles were found in the original search using the terms “India”, “diet”, and “nutrition assessment”. Web of Science yielded 17 articles from the search terms “India”, “diet”, and “nutritional assessment”. Furthermore, 80 articles were found on Embase using the search terms “India”, “dietary intake”, and “assessment”. In total from these three databases, 159 articles were found. After removing duplicates using Mendeley, there were 148 articles shortlisted.

### Literature Selection

Selected studies were limited to nutrition assessment of children and adolescent. Only publications in English language were selected. The inclusion criteria for the review articles selected were that the study 1) was conducted in an Indian population, 2) included methodology for dietary assessment, and 3) reported dietary intake at the nutrient level i.e., report of either a single nutrient, including calories, macronutrients or micronutrients. The exclusion criteria were 1) studies on food composition not collected from human subjects, 2) studies that reported dietary intake at the food or food group level.

### Outcome Measures

The outcome of interest was report of dietary consumption at the nutrient level for a specific dietary assessment method. In addition, challenges and opportunity related to dietary assessment were also included. The objective was to understand the types of dietary assessment tools and methods used in different Indian populations. The cost of food in a diet was not included as outcome.

### Data Selection and Extraction

Every title and abstract were reviewed by two reviewers to determine which review articles fulfilled the criteria above. If the criteria above were fulfilled, or it could not be determined whether the criteria was fulfilled in the abstract alone, the full article was exported for manual review. Overall, 67 articles met these criteria and were read in full to determine if they should be included in the final table. **Table 3** includes all 41 articles that fulfilled the search criteria. Articles were excluded from the table if they did not analyze nutrients specifically, only looked at dietary diversity rather than nutrient intake, the nutrient composition of food was determined in a lab, not by dietary intake, or they could not be accessed. Thus, data was extracted manually by reading each article. Numeric data such a percentage, counts etc. were manually extracted in put in a table format.

### Search and Selection

**Figure 1:**
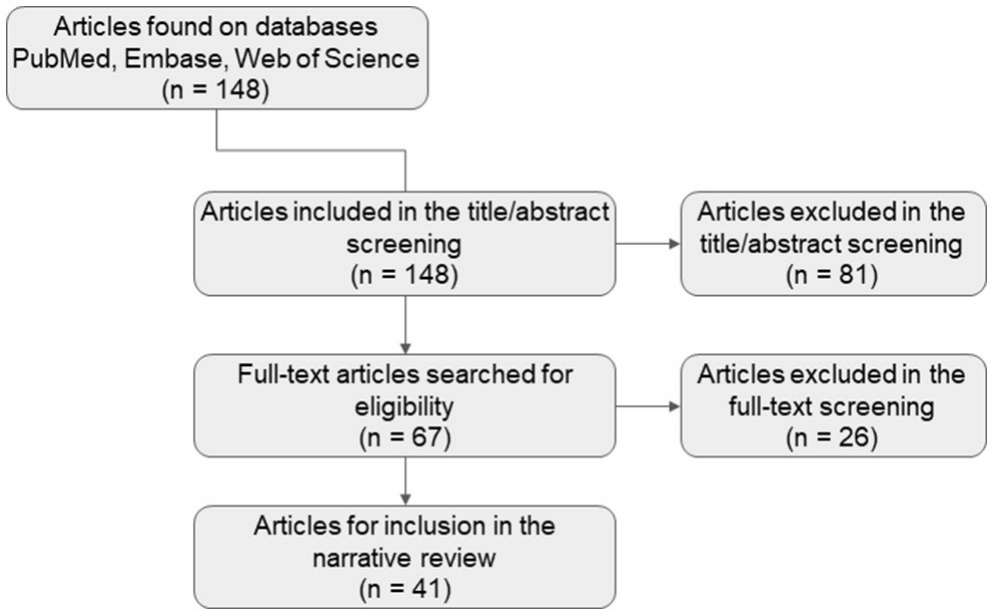
Literature search process diagram.

Figure 1 summarizes the search strategy for articles that provided quantitative dietary intake information in Indian participants. In total, 148 articles were found were found from different online databases and after title and abstract screening 81 were excluded from the searched dataset. The 67 full-text publications were screened in detail against the prospectively defined inclusion / exclusion criteria and 41 studies included in this analysis, with 26 excluded. Articles were excluded from the table if they included laboratory techniques for analyzing nutrient composition of food items or if dietary intake was summarized on a broad level (such as food groups, diversity, etc.), and did not quantitatively estimate nutrient intake.

### Risk of Bias

Quality assessment of the articles was independently performed by two reviewers and disagreements were addressed before final selection to be included in this manuscript.

## Discussion

### Dietary Recommendations for Children and Adolescents

Children and adolescents have unique macronutrient and micronutrient needs compared to adults, and their nutritional needs change across the phases of development. Their risk factors for sub-optimal diets also change as children age (4, 6). For example, during infancy and early childhood, children are almost entirely dependent on their caregivers; however, as children age, they are increasingly exposed to food environments in schools, marketing and advertising, and peer pressure and changing taste preferences (4). The World Health Organization (WHO) recommends exclusive breastfeeding for infants during their in first 6-months after birth (20). From 6 to 24 months, the WHO recommends continued breastfeeding along with diverse and nutrient-dense complimentary food. After 2 years, children and adolescents are recommended to consume nutrient-dense foods from 4–5 food groups per day with increasing caloric needs as children and adolescents continue to grow. From 2 years, children should consume 1,400–1700kcal/day; by middle childhood, caloric needs reach 1500–2100 kcal/day, and by 10 years, children and adolescents are recommended to consume 2000–3300 kcal/day. The WHO also recommends limits on the daily intake of foods with added salt, sugar and fat among across all age groups. The Indian National Institute of Nutrition (NIN) recently published a short report on the nutrient requirements for Indians, including the Estimated Average Rrequirements (EAR) and Recommended Daily Allowances (RDAs) for Indian children and adolescents (21). The EAR is the median usual intake value that is estimated to meet the requirements of half the healthy individuals in a life stage and gender group, while the RDA estimates the average daily level of intake sufficient to meet the nutrient requirements of nearly all (97%–98%) healthy people in the specified age and gender group.

### Current State of Knowledge of Diets of Children and Adolescents in India

The importance of high-quality diets for children and adolescents in India is well recognized. However, the data on whether young people are achieving the recommended dietary targets is limited to sub-national surveys and research studies in different targeted populations using non-standardized methods, making comparison of dietary targets problematic. Often key nutrition indicators, including some indicators of child or adolescent diet are not the focus of the surveys, but are collected as a part of large surveys that assess vital statistics for the risks and prevalence of various health challenges in India (Table 1). For example, the cross-sectional National Family Health Survey (NFHS), conducted every 4–10 years, includes a nationally representative sample to provide data on several health indicators, but does not include direct, individual-level dietary assessment. Nutrition-related indictors collected in children and adolescents include: anthropometry, infant feeding practices, hemoglobin assessment, household use of iodized salt and vitamin A supplementation coverage. The NFHS-4 found that in 2016, among children under 5 years: 38% were stunted, 21% were wasted and 2% were overweight, and 58% of children 6–59months were anemic. Data on adolescents are not presented independently, but 21% of women and 19% of men from 15–49 were overweight or obese and 53% of women aged 15–49 were anemic and 23% had BMI <18. The Comprehensive National Nutritional Survey (CNNS: 2016–2018) supplements the NFHS with detailed micronutrient assessments in children and adolescents aged 0–19 years. It found that the prevalence of anemia was 41% among pre-school age children, 24% in school-aged children and 28% in adolescents. The prevalence of zinc deficiency was 19% in preschool children, 17% in school-age children and 32% of adolescents. Additionally, 18% of pre-school children, 22% of school-age children and 16% of adolescents were identified to have low serum retinol levels indicating vitamin A deficiency (22). The National Nutrition Monitoring Bureau (NNMB) conducts surveys with detailed dietary assessments; however, it does not conduct surveys in nationally representative samples, but rather targets selected states and cities within the country. Regardless, the NNMB surveys provide key information on the diet and nutrition of selected areas and also provide data to evaluate ongoing national nutrition programmes, such as the mid-day meal programme and the national nutrition anemia control programme. In addition to anthropometric, clinical and hematological parameters, the NNMB collects 24 h recalls and weighed diet surveys in selected sub-samples to describe the food groups consumed as well as the macronutrient and micronutrient intakes of targeted populations. NNMB data highlights key gaps in the diets of children and adults throughout India. Notably, in the 2017 Report for Nutritional Status of Urban Indian population reported that among children age 1–3 years, the average caloric intake was 55.5% of the RDA for this age group, 36.5% for iron and 15% for vitamin A (23).

**Table 1:** National & sub-national surveys that assess the nutrition of children & adolescents in India.

**Survey**	**Sample**	**Sample size & sampling frame**	**Nutritional assessment methods**	**Recent national estimates**
NFHS-4, 2015–16	National	*n* = 219,796 (<5yrs) 2-stage sampling: PPS to select villages; random selection of households w/in villages	Anthropometry: HAZ, WHZ, WAZ Biochem: Hb Diet: IYCF	<5yrs: 38% stunt., 21% wast., 36% undrwt, 2% ovrwt, 58% anemic (6–59mo)
CNNS, 2016-18	National	*n* = 38,060 (<5 yrs) *n* = 38,355 (5–9 yrs) *n* = 35,830 (10–19 yrs) Multi-stage sampling: PPS to select PSUs, systematic selection of households from each PSU, then segmentation to reduce sample size	Anthropometry: HAZ, WHZ, WAZ, BMIZ, circumferences, skin folds Biochem: Hb, Serum ferritin, retinol, 25(OH)D conc., zinc, B12, erythrocyte folate Diet: IYCF, dietary diversity	<5yrs: 35% stunt., 17% wast., 33% undrwt, 2% ovrwt, 41% anemia (6–59mo), deficiencies: 18% VAD, 23% folate, 14% B12, 14% VDD, 17% Zinc 5–9yrs: 22% stunt., 10% undrwt, 23% thin., 24% anemic, deficiencies: 22% VAD, 28% folate, 17% B12, 18% VDD, 17% Zinc 10–19yrs: 5% undrwt, 24% thin., 28% anemic, deficiencies: 16% VAD, 37% folate, 31 % B12, 24% VDD, 32% Zinc
Rapid Survey on Children, 2013–14	National	*n* = 83,324 (<5yrs); *n* = 25,186 (girls 10–14 yrs); *n* = 20,374 (girls 15–18 yrs) Multi-stage stratified random sampling. Villages at PSU and Households as SSU	Anthropometry: HAZ, WHZ, WAZ, BMIZ Diet: IYCF	<5yrs: 39% stunt., 15% wast., 29% undrwt IYCF 6–23mo: 41% MMF, 11% MAD, 22% MDD Girls 10–18yrs: 63% thin, 2% overweight, 1% obese
NNMB surveys (Urban 2015–2016; Rural 2011–2012; Tribal 2007–2008)	Sub-national	NNMB Urban 2015–2016 survey included: *n* = 12,097 (<5yrs) & 36,656 (5–17yrs) Multistage random sampling: 5 cities from 16 states, random sampling at municipal ward level	Anthropometry: HAZ, WHZ, WAZ, circumferences, skin folds Diet: 24 h rec., FFQ & IYCF	No national estimates
Annual Health Survey-CAB, 2014	Sub-national	Sample size varies by state & assessment Stratified simple random sampling. The sample units are Census Enumeration Blocks (CEBs) in urban areas and villages in rural area.	Anthropometry: HAZ, WHZ, WAZ Biochem.: Hb Diet: Iodized salt consumption	No national estimates

The COVID-19 pandemic and its economic, political and food security effects have drastically impacted nutrition security including a decline in household food expenditure and limited availability of diverse food items. Surveys show that during the pandemic, consumption of eggs, meat, vegetables and fruits declined significantly in multiple districts throughout India (24). At the same time, Low and Middle Income Countries experienced an estimated increase in the prevalence of wasting in children from 18 to 23 percent (25). In India, specifically, it is estimated that the pandemic and subsequent economic impacts could contribute to an additional 410,413 cases of underweight and 392,886 cases of wasting among young children above the estimates from the 2015–2016 NFHS (26).

### Overview of Methods for Measuring Dietary Intake

There are several different ways to measure nutritional status. Indicators of food intake can be divided into direct and indirect measures. Indirect measures of food intake use data on food availability at the national, regional or household level to estimate intake, rather than directly collecting information from individuals (27). Common sources of data for indirect measurement include national commodity-level food supplies, such as the Food and Agriculture Organization (FAO) balance sheets and household surveys that assess indicators such as household expenditures, procurement, and food inventory. In India, the National Sample Survey (NSS) and the India Human Development Survey (IHDS) provide information on uncooked food consumption pattern of households, which provides information on household food security, but do not directly assess the diets of the children or adolescents in the family (28). A recently developed indirect nutritional assessment approach of particular relevance to children and adolescents is the Comprehensive Nutrient Gap Assessment (CONGA), based on the theory of health implications of nutrient deficiencies at both the individual and population levels. This method provides guidance on how to assess nutrient gaps from evidence from five different sources i.e., i) biological, clinical, and functional markers, ii) nutrient adequacy of individual diets (modeled using food composition and requirement data), iii) nutrient adequacy of household diets (modeled using food composition and requirement data), iv) nutrient adequacy of national food supplies (modeled using food composition and requirement data), and v) nutrient-informative food group intake of individuals or households (29).

Acknowledging the need for targeted interventions in the LMIC's, where affordability is a key factor influencing the food choices, the CONGA approach considers the population specific data along with the demographic data and identify gaps for prioritized nutrients and rate them from negligible to high in terms of severity. Based on this method, nutrient gaps in Indian children aged 6–23 months have been identified as high for iron, zinc and folate, and moderate for vitamin A, vitamin B12 and calcium, and key foods, such as organ meats, other animal products and green leafy vegetables are proposed to fill many of these gaps (30).

Each nutrition and food security indicator provides a different piece of socioeconomic and health information. The indirect measures described above are useful to assess the accessibility and availability of food, and are essential to support public health policies relating to food security and nutrition among children and adolescents in LMIC's (31); however, direct measures of food intake are required to estimate individual-level dietary intake, which is of particular interest when studying the nutrition of children and adolescents. One way to categorize individual-level nutritional assessment methods is the “ABCDs”: anthropometry, biochemical assessments, clinical assessments and dietary assessment (27). The latter, which is the focus of this manuscript, is particularly complex to measure in free-living individuals given the frequency with which people consume foods and beverages throughout each day, the day-to-day variation in diet, and the dependence of researchers on participants engaging in burdensome, prospective recording as consumption occurs or on participant recall. The challenges of dietary data collection are further compounded by the need to adapt measures and methods to local diet and food practices in different contexts (32). Broadly, the methods of directly measuring individual-level dietary intake can be divided into two categories: 1) Non-quantitative methods such as diet quality screeners or questionnaires designed to provide summary indicators that summarize aspects of diet quality of a particular parameters of interest such as the dietary diversity, or proportional consumption based on food groups. However, non-quantitative methods cannot be used to quantify consumption of nutrients; and 2) quantitative methods that collect detailed dietary data, including portion sizes and recipes. Quantitative data can be used to estimate intake of calories, macronutrients and micronutrients.

### Summary Indicators and Diet Quality Screeners

There are several dimensions of diet quality including: adequacy, nutrient density, macronutrient balance, diversity and proportionality, the avoidance of excess consumption, food safety, and sustainability (33). Summary metrics of diet quality that are commonly used to assess the diet of children and adolescents include the WHO/UNICEF IYCF indicators, which are the most commonly used indicators for tracking IYCF trends over time, targeting and monitoring and evaluation. Most existing research utilizes the 2007/2008 WHO/UNICEF indicators which included 8 core indicators and additional optional indicators. The 8 core indicators are commonly assessed in national surveys and research studies in India, and globally. They are: (1) early initiation of breastfeeding; (2) exclusive breastfeeding under six months; (3) continued breastfeeding for one year; (4) the introduction of solid, semi-solid or soft foods; (5) minimum dietary diversity; (6) minimum meal frequency; (7) minimum acceptable diet; and (8) consumption of iron-rich or iron fortified foods. The new 2021 WHO/UNICEF Indicators for assessing IYCF practices recommend 17 indicators including consumption of unhealthy foods and zero fruit or vegetable consumption, though to date, these indicators have not yet been broadly widely adopted.

Dietary diversity is also frequently measured in women of reproductive age (age 15–49 years) with the minimum dietary diversity score for women (MDD-W) (34). The IYCF indicators and the MDD-W can both be conducted relatively quickly, and can thus be embedded into large-scale surveys. It is important to note, however, that dietary diversity metrics for women and for children 6–23 months were designed and validated for their correlations with the probability of adequate micronutrient intake. They cannot assess other dimensions of diet quality, and they do not quantitatively measure nutrients consumed. Moreover, these diversity measures were designed and validated to be used as population measures, not as individual measures. There are several diet quality screeners that have been designed to quickly gather diet quality information from participants without quantitative assessment. These include the short Diet Quality Screener (sDQS) (35), the KidMED (36) and the Prime Diet Quality screener (PDQS) (37). Most diet quality screeners aim to assess aspects of diet that have been shown to be associated with that have cardio metabolic outcomes, and were developed based on the diets of adults in high-income countries (33). Research on the development of simplified measures of diet quality that can be used in different populations in LMICs is ongoing, with current attention focused on women of reproductive age (33). However, there is a great need for simplified questionnaires and metrics that can describe dimensions of dietary quality beyond micronutrient deficiencies, and are also appropriate for use in children older than 2 years and in adolescent males.

### Quantitative Dietary Assessment Methods

Detailed, quantitative individual intake assessment is necessary to describe the actual nutrient intake of children and adolescents in greater detail beyond what simplified metrics can include. There are several methods for directly assessing individual dietary intake including: 24 h dietary recalls, food frequency questionnaires (FFQs), food diaries, and weighed diet records (38). These methods provide quantitative data that can be used to estimate the intake of specific nutrients and dietary components (27, 32). In summary, all of these methods include the following steps: 1) obtain a report of all of the foods consumed by an individual over a specified period of time; 2) identify the foods in sufficient detail to link it to a corresponding food table; 3) quantify portion sizes and frequency of consumption; and 4) calculate nutrient intake from a food table. The current time and cost burdens of the methods of traditional dietary assessment are substantial. One study, which examined the costs of 24 h recalls in households in sub-Saharan Africa and South Asia estimated the average cost to be $247 per household (39). However, notably, there is limited research on the true cost of conducting and analyzing dietary data.

In summary, large scale surveys provide essential overviews of nutrition-related indicators in children and adolescents in India that should be used to shape national and local policies; however, detailed, individual-level quantitative dietary data collection has been completed by only a handful of research teams in non-representative populations. As part of this review, we systematically reviewed the existing peer-reviewed scientific literature with quantitative dietary data collection in order to determine what methods and tools individual research teams are using to conduct dietary data collection in India.

### Unique Challenges of Measuring Dietary Intake in Children and Adolescents

The most important factor for accurate dietary assessment is the active participation of the respondent. When the respondents are children and adolescents, it creates additional challenges for dietary assessment because of their changing cognitive capacity and reliance on others for feeding and care practices. Thus, until children reach their full cognitive awareness of their food intake, the recall for the younger age aged between 0–7 years is mainly dependent on the parents/care-takers (40). The challenges with respect to dietary assessment in children include parental dietary bias during dietary recall, inaccurate memory, misreporting and any illness episode when data was being collected, that can lead to either over or under estimating the child's intake by the parent. Studies in high income country contexts have shown that parents or care-takers can accurately report the at-home intake of children (38, 41, 42). However, the meals eaten by a child outside their home may be under-reported, particularly as children age and begin to consume more of their meals away from their primary caregiver. Research has also indicated that around 7–8 years of age, children can self-report intake, but only for the previous 24 h, thus making longer-term methods, such as Food Frequency Questionnaires (FFQ) inappropriate in this age groups (40). Even with short-term recalls, young children often lack comprehension of portion size, frequency of intake, detailed identification of the food type, which can results in a lack of precision (43). For adolescents, new challenges in collecting dietary intake data arise due to increased consumption of meals outside the home, increased variability in the foods consumed, changes in social desirability in reporting and potentially a decrease in co-operation and motivation (40). One potential strategy to improve assessment of dietary intake in older children and adolescents is to combine their dietary recall with additional data collected from parents (44, 45); however to date, there is no clear consensus on which parameters should be collected from parents and/or how to efficiently implement this dual-reporter process. Research on how to accurately collect quantitative dietary data in children and adolescents, particularly in LMICs is sorely needed. Without it, larger trends in dietary patterns—such as low fruit and vegetable intake or high intake of processed foods—can continue to be monitored, but accurate information on child and adolescent nutrient intake will continue to be a major challenge (6).

### Studies Assessing Nutrient Consumption in India

Our literature search yielded 41 articles that collected quantitative dietary intake data from participants in India. Majority of the studies were conducted in the North Indian regions (44%), while the remaining regions contributed to 20% (*n* = 8) each of the total 41 studies of all the studies. A total of 41 articles were included in Table 2, which summarizes the various dietary and nutritional assessment methods/tools adopted by researchers in India. The majority of these studies assessed nutrient intake in adults only (*n* = 27) (19, 49, 51–64, 66–72, 74), with fewer studies that included assessment in children (*n* = 12) (8–17, 46, 47), and adolescents (*n* = 4) (18, 48–50). The most common methods of dietary assessment were 24-hr recalls and FFQs: 36 studies used a 24 h recall and 10 used FFQs that were designed by individual research teams for their study sample based on 24 h recalls or focus group discussions.

**Table 2:** Studies in India Assessing Nutrient Intake and Assessment Method.

**Author, Year**	**Study sample size, age and location**	**Assessment method**
**Studies including children & adolescents**		
Singh et al. (9)	Children (0–5 yrs), *n* = 914, Rajasthan	24 hr recall
Bains et al. (10)	Children (6–59 mos), *n* = 312, Punjab	24-hr recall
Nithya et al. (11)	Children (1–5 yrs), *n* = 344, Odisha	24-hr recall, FFQ
Manu et al. (12)	Children (3–4 yrs), Haryana	24-hr recall
Sharma et al. (13)	Children (3 −5 yrs), *n* = 150, Odisha	24-hr recall, FFQ
Chyne et al. (8)	Children (<5 yrs) *n* = 603 & mothers, *n* = 500, Meghalaya	24-hr recall
Sivaramakrishnan et al. (46)	Children (≥8yrs) & adults, *n* = 1027, Maharashtra	24-hr recall
Mitra et al. (14)	Children & Adolescents (4–12 yrs), *n* = 309, Chhatisgarh	24-hr recall
Loukrakpam et al. (15)	Children & adolescents (5–17yrs), women (18–49yrs) *n* = 690, Manipur	24-hr recall
Basu et al. (47)	Children, Adolescents & Adults, *n* = 405, West Bengal	24-hr recall
Chiplonkar et al. (16)	Adolescents (10–16 yrs), *n* = 630, Maharashtra	24-hr recall
Malhotra et al. (17)	Adolescents (11–12 yrs), *n* = 209, Uttar Pradesh	24-hr recall, FFQ
Jeyakumar et al. (18)	Adolescents (16–18 yrs), *n* = 565, Maharashtra	24-hr recall
Ghosh-Jerath et al. (48)	Adolescents and Adults, (15–49 yrs), *n* = 151, Jharkhad	24-hr recall
Ghosh-Jerath et al. (49)	Adolescents and Adult women (15–54 yrs), *n* = 143, Jharkhand	24-hr recall, FFQ
Menon et al. (50)	Adolescents & Adults, (≥15yrs), *n* = 363 yrs, South India	1-week diet recall
Deb et al. (51)	Adults (15 + yrs), *n* = 208, West Bengal	24-hr recall
**Studies in Adults Only**		
Pathak et al. (19)	Women (18 yrs), *n* = 288, Haryana	24-hr recall
Vijay et al. (52)	Adults (18+ yrs), *n* = 460, Kerala	FFQ
Swaminathan et al. (53)	Adults (18+ yrs), *n* = 636, Chennai & Madhurai	24-hr recall
Hebert et al. (54)	Adults (18+ yrs), *n* = 120, Kerala & Gujarat	24-hr recall
Bhatt et al. (20)	Adults (18+ yrs), *n* = 342, New Delhi	24-hr recall, FFQ
Bellows et al. (55)	Pregnant women (18+ yrs), *n* = 627, Uttar Pradesh	24-hr recall
Rao et al. (56)	Pregnant women (18+ yrs), *n* = 41, Maharashtra	24-hr recall
Gautam et al. (57)	Pregnant women (18+ yrs), *n* = 116, New Delhi	24-hr recall, FFQ
Sathiaraj et al. (58)	Adults (18+ yrs), *n* = 260, Tamil Nadu	FFQ
Singh et al. (59)	Women (18+ yrs), *n* = 90, Rajasthan	24-hr recall
Agrahar-Murugkar et al. (60)	Women, (18–50 yrs), *n* = 650, Meghalaya	24-hr recall, FFQ
Venkatramanan et al. (61)	Women, (18–55 yrs), *n* = 126, West Bengal	24-hr rec., FFQ, weighed lunch
Misra et al. (62)	Adults, (18–69 yrs), *n* = 227, New Delhi	24-hr recall, FFQ
Subasinghe et al. (63)	Adults, (19–85 yrs), *n* = 45, Andhra Pradesh	24-hr recall, weighed record
Pai et al. (64)	Adults (20+ yrs), *n* = 420, Karnataka	24-hr recall
Sudha, V et al. (65)	Adults, (≥20 yrs), South India	24-hr recall, FFQ
Porkharel et al. (66)	Adults (20–55 yrs), *n* = 66, Udupi	24-hr recall, FFQ
Sivaprasad et al. (67)	Adults, (21–60 yrs), *n* = 630, South India	24-hr recall
Mahalle et al. (68)	Adults (25–92 yrs), *n* = 300, Maharashtra	24-hr recall
Daniel et al. (69)	Adults (35–69 yrs), *n* = 3908, Maharashtra & Kerala	24-hr rec., custom quest.
Agarwal et al. (70)	Women (60–85 yrs), *n* = 50, Gujarat	24-hr recall
Natarajan et al. (71)	Adults (60+ yrs), *n* = 420, South India	3-day diet recall
Gupta, A et al. (72)	Adults (60 + yrs), *n* = 255, Uttarakhand	24-hr recall, FFQ
Prasad et al. (73)	Adults, *n* = 283, Lucknow	3-day diet diary

### Existing Tools to Analyze Dietary Data to Estimate Nutrient Intake

One of the most challenging parts of dietary assessment is to estimate nutrient intake from dietary data. This requires that each food item consumed by a participant be matched with a locally appropriate food table that has data available on ingredients, preparation methods or recipes, conversion factors and nutrient values for each ingredient. Several data sources provide the nutritive value of foods in the Indian diet, including the 2017 Indian Food Composition Table (IFCT) (75), the Nutritive Value of Indian Foods (ICMR) (76) and the National Institute of Nutrition (NIN) book of Some Common Indian Recipes and their Nutritive Value (77). The IFCT includes 160 food constituents (macronutrients, micronutrients & bioactive compounds) analyzed for 528 predominantly raw food items, while the NIN and ICMR sources add additional nutritive values, particularly for cooked items and mixed dishes. Given the wide variability in cooking and dietary practices across India, these sources do not comprehensively include all of the foods consumed throughout the country. Although these food composition and recipe resources are an essential first step in analyzing dietary data, key barriers in utilizing these resources for large-scale epidemiologic studies, include: 1) the need to collect local recipes and conversion factors for each new geographic area of study, and 2) software to smoothly link a large number of dietary intakes (often in the hundreds or thousands) to local recipe databases and national food composition tables.

Due to the lack of appropriate local food and recipe composition databases and the need to manually clean, code and analyze each intake, many researchers often skip the final step in quantitative dietary assessment (calculation of nutrient intakes), and instead choose to describe the data by food groups avoiding the task of estimating calorie, macronutrient and micronutrient consumption. The 41 studies in Table 3 are the only studies that we identified that completed the final step of nutrient intake estimation. Table 3 summarizes the findings from the twelve studies that estimated nutrient intake in children or adolescents. For comparability, a study in patients with epilepsy (including adults) (50) and a study on breakfast behaviors in children from high and middle-income households (46) were excluded. Research methodologies and study samples varied across studies; however, across all studies dietary intakes of macronutrients and micronutrients were below the RDA in each sample studies.

**Table 3:** Studies assessing nutrient intake of Children and Adolescents (0–18yrs) in India.

**Author**	**Sample**	**Nutrient intakes reported:**	**Key findings**
Chyne et al. (8)	Children (<5 yrs) *n* = 603 & mothers, *n* = 500, Meghalaya	Energy, protein, fat, calcium, iron, phosp., vit A, vit C, folate, thiamine, riboflavin, niacin	The mean intake below RDA for: Energy, protein, fat, calcium, iron, phosphorous, vit A, vit C, folate, Thiamine, Riboflavin, Niacin
Singh et al. (9)	Children (<5 yrs), *n* = 914, Rajasthan (drought-affected population)	Energy, protein	Mean intake below RDA for: Energy, protein
Bains et al. (10)	Children (6–59 mos), *n* = 312, Punjab	Energy, protein, carbs., fat, fibers, calcium, iron, zinc, vits A, B12, B6, C, folate, thiamine, riboflavin, niacin	Mean intake below RDA for: cal., protein, calcium, iron, zinc, vit C, folate, riboflavin, B6 & niacin
Nithya et al. (11)	Children (1–5 yrs), *n* = 344, Odisha	Energy, protein, fat, calcium, iron, vit A, vit C, folate, thiamine, riboflavin, niacin	Average food scores & DDI <70% NAR for fat, calcium, iron, vit A, vit C, folate, vit B9, thiamine, riboflavin, niacin
Manu et al. (12)	Children (3–4 yrs), Haryana	Energy, protein, fat, calcium, iron, vit A, vit B12, vit C, folic acid, thiamine, riboflavin, niacin	The mean intake below RDA for: Iron and vitamin C
Sharma et al. (13)	Children (3–5 yrs), *n* = 150, Odisha	Energy, protein, fat, calc., iron, zinc, vit C, folate	The mean inake below RDA for: Energy, calcium, iron, zinc, vit C
Mitra et al. (14)	Children & adolescents (4–12 yrs), *n* = 309, Chhatisgarh	Energy, protein	The mean intake below RDA: Energy, protein
Loukrakpam et al. (15)	Children & adolescents (5–17yrs), women (18–49yrs) *n* = 690, Manipur	Energy, protein, carbohydrates, fat, fibers, calcium, iron, phosphorous, sodium, zinc, vit A, vit B1, vit B2, vit B3, vit B6, vit C, vit E, folate	The mean intake compared to AMDR: Macronutrient consumption was less than AMDR The MPA (<0.5) for: Calcium, vit A, Vit C
Chiplonkar et al. (16)	Adolescents (10–16 yrs), *n* = 630, Maharashtra	Energy, protein, carbohydrates, fat, fibers, phytate, calcium, copper, iron, zinc, beta carotene, vit C, riboflavin, folic acid	The mean intake below Indian RDI: Energy, protein, carbohydrates, fat, fibers, phytate, calcium, copper, iron, zinc, beta carotene, vit C, riboflavin, folic acid
Malhotra et al. (17)	Adolescents (11–12 yrs), *n* = 209, Uttar Pradesh	Energy, protein, calcium, iron, Vit A, Vit C, folic acid, niacin, riboflavin, thiamin	The mean intake below RDA: Energy, vit A, Folic acid, Iron
Jeyakumar et al. (18)	Adolescents (16–18 yrs), *n* = 565, Maharashtra	Energy, fat, protein, fibre, iron. Beta-carotene, vit C, folate	The mean intake below RDA: Energy, protein, Iron for anemic girls
Pathak et al. (19)	Women (16+ yrs), *n* = 288, Haryana	Energy, protein, iron, retinol	The mean intake below RDA: Energy, protein, iron, retinol

Notably, the majority of studies listed in Table 3 did not describe their methods for linking their intake data to databases to estimate nutrient intakes. This is consistent with the observation that dietary research studies in other LMIC contexts usually complete the collection, entering, cleaning, processing and dietary data analysis manually (7). This process is incredibly time-consuming and expensive (7, 39) and may introduce substantial systematic errors (32). A handful of the Indian studies reviewed describe the use of commercial software platforms (13, 48, 53, 68, 78) or the development of in-house databases (69) to analyze nutrient intakes. The creation of study-specific software platforms for the collection and analysis of dietary data (69, 79) can improve efficiency and minimize errors introduced by the inclusion of multiple data coders. However, the need for each research team to independently develop new platforms for each study or catchment area requires substantial technical experience that is beyond the means of most research projects.

### New Technologies to Collect and Analyze Dietary Data in India

Public-private partnerships may provide important opportunities for nutritional epidemiologists in India. Theoretically, electronic data capture systems that are used for mobile health projects could be adapted for dietary data collection, such as COMM Care, ODK Collect, Pedragon, Mobile InterVA (MIVA) (7, 80); however, in their current form, the use of these platforms for dietary data collection involves substantial preparation from research teams to customize and prepare these platforms (including pre-coding food items, collecting recipes and conversion factors, and preparing portion size guides) to ultimately link the collected data to food composition databases (69, 79). The few existing 24 h recall data collection platforms, such as ASA24 and GloboDiet, were designed for use in high-income settings (81, 82).

Recently, online applications designed for individual consumers, nutritionists and business to assess individual dietary intake have emerged in India. These types of platforms have the potential to be further developed to support epidemiologic research in nutrition in India. For example, the DietSoft (Invincible Ideas, Noida, Uttar Pradesh, India) software for dietary calculations is targeted toward clinicians or businesses (restaurants, hotels, etc.) to estimate the nutritive value of 24 h recalls, custom recipes, and other food intakes (83). The database includes 1,205 common Indian food items including all foods in the India Food Composition Tables (IFCT) (75), the National Institute of Nutrition (NIN) Nutritive Value of Indian Foods list (76) and brand-name items. Researchers can also add any locally-collected recipes or food items. Several challenges would need to be addressed in order for the software to be used for epidemiologic studies, including the ability to organize data for large-scale epidemiologic data as opposed to summarizing individual intakes. The Ntuitive software is also designed for use by Indian dieticians and nutritionists to plan personalized diet plans for their patients. The software can collect individual level dietary data, build a personalized recipe database, and perform nutritional analyses of diet intake and diet plans based on a 8,000 item food database which includes the IFCT foods as well as culturally diverse recipes from different regions of India. Similar to DietSoft, however, the software is designed to collect individual level data, and would need to be substantially revamped to aggregate data at population level, and hence in its current form, the application of this software for public health research is limited.

Additional software platforms developed for global contexts may soon be available for assessing the diets of children, adolescents and adults in India in the near future. For example, the INDDEX24 platform (7) is currently being validated in a handful of LMICs, with some early adopters incorporating its use in large surveys and research studies (personal communication with the INDDEX24 team). INDDEX24 aims to integrate dietary reference data management and reporting tools with an easy-to-use, mobile, quantitative 24 h dietary survey tool. The platform aims to be adaptable to new settings in LMICs, and in particular aims to link to a Global Food Matters Database—an open-source food database with items and recipes from around the world. In the future, INDDEX24 could potentially integrate with existing Indian resources to increase the ease and feasibility of conducting 24 h recalls in children and adolescents in India. However, to date, Global Food Matters Database does not include the IFCT or other Indian food composition databases. An alternative global application is the Global Dietary Quality Score application, which will collect dietary data specifically for the calculation of a diet quality score (84). The score factors in the consumption of both healthy and unhealthy food groups and has been shown to be associated with nutrient adequacy and with several NCD risk markers in Indian women (85). Preliminary research on the GDQS calculates the score from 24 h recalls or FFQs; however, a free application is currently under development for use by researchers and program implementers (84). The application will rely on the collection of dietary data through a multiple pass method, similar to the USDA 24 h recall multi-pass method (86). After collecting quantitative information on the consumption of 25 food groups, the application will generate a GDQ score, and will describe food group consumption, but is not designed to estimate nutrient intakes. These two applications have notable potential for streamlining dietary data collection in India; however, both the INDDEX24 and the GDQS applications are currently undergoing development. Neither application has yet been tailored for use in India, and notably the GDQS has not yet been validated for use in children.

In addition to increasing nutrition insecurity globally, the COVID-19 pandemic has also highlighted the value of research assessment methods that can be completed over the phone or through the use of a mobile application, as opposed to in-person, interviewer administered questionnaires. Several researchers from high-income countries have utilized and validated telephone and online surveys for dietary data collection (87–90). However, telephone and mobile application data collection, and the specific applications used, need to be validated in different contexts to ensure high-quality dietary data collection. Several Indian research organizations have adapted to the telephonic surveys for the purpose of data collection in adults during the pandemic (85, 91); but to date, there are no studies that have utilized telephone or computer-based systems for dietary data collection with validated results. It is possible that some of the online dietary data collection tools described above could be adapted for self-administration among literate adolescents or parents of young children; however, at this stage, additional investment and research on how to transition to telephone and/or self-administered online applications is much needed.

## Conclusion

Summary indicators of diet quality are currently the only practical method for describing the diets of children and adolescents in India and other LMICs at scale. However, there is an important need to continue research into how to improve these summary indicators—such as developing and validating indicators for older children and male adolescents, and the capturing of aspects of diet quality in addition to diet diversity (6, 33). Investments should also be made to improve the collection of individual-level quantitative dietary data in India that would streamline the estimation of nutrient intake among children and adolescents, but also adults. Collecting individual-level quantitative dietary data in India is currently an expensive and time-consuming process (7). However, comprehensive data on nutrient intake can improve the planning of and evaluation of effective policies and interventions targeting the dietary habits in populations with unique nutritional needs. Increased government, private and donor investment in dietary intake research in India could smooth several of the bottlenecks described in this review. Research should specifically be conducted on the development and validation of novel approaches to dietary data collection in children and adolescents, given the unique challenges of collecting accurate data from these populations (38, 40). In addition, incentives and organized platforms for sharing resources have the potential to promote collaboration and information sharing in order to prevent the constant re-creation of local recipe and food databases. In the absence of a centralized structure for sharing the inputs required for dietary assessment such as: lists of commonly consumed items, detailed data from local recipe collections, nutrient composition of brand name food items, portion conversion factors, and yield and retention factors; researchers will continue to be forced to reproduce these items for each new assessment.

The Indian National Nutrition Strategy's 2022 vision aims to ensure that every child, adolescent girl and woman attains optimal nutritional status. As part of this strategy, India's Nutrition Programmes such as the Integrated Child Development Services (ICDS), Mid-Day Meal scheme, POSHAN Abhiyan and Anemia Mukt Bharat programs play a key role in protecting the nutritional status of women and children throughout the country. Additionally, the Rashtriya Kishor Swasthya Karyakram supports the nutrition of Indian adolescents. Strengthened direct investment in adolescent and child health will have a significantly positive and direct impact on the India's development (92). Notably, many individual nutrition outcomes can be achieved without changing and measuring dietary habits, such as the promotion of breastfeeding or the implementation of micronutrient supplementation and fortification programs. However, addressing the triple burden of malnutrition in India in a sustainable and holistic way will require interventions aimed and improving the dietary patterns and behaviors of India's youth. As Lord Kelvin, the famous 19th century physicist aptly proclaimed, “if you cannot measure it, you cannot improve it.” Food consumption and healthy diets are at the crux of the intersection between the policy goals of the nutrition and food security fields of study (93). Improving the nutrition and health of India's children and adolescents will require renewed investments in improving dietary quality, and supporting the measurement of diet.

## Author Contributions

LL and MS wrote the first draft of the manuscript. All authors wrote sections of the manuscript and contributed to manuscript revision, read, and approved the submitted version.

## Conflict of Interest

The authors declare that the research was conducted in the absence of any commercial or financial relationships that could be construed as a potential conflict of interest.

## Publisher's Note

All claims expressed in this article are solely those of the authors and do not necessarily represent those of their affiliated organizations, or those of the publisher, the editors and the reviewers. Any product that may be evaluated in this article, or claim that may be made by its manufacturer, is not guaranteed or endorsed by the publisher.
